# Characterisation of mexiletine’s translational therapeutic index for suppression of ischaemia-induced ventricular fibrillation in the rat isolated heart

**DOI:** 10.1038/s41598-020-65190-y

**Published:** 2020-05-21

**Authors:** Louise M. Hesketh, Catherine D. E. Wilder, Niraja N. Ranadive, Georgia Lytra, Patrisia Qazimi, Jade S. Munro, Nakita Ahdi, Michael J. Curtis

**Affiliations:** Cardiovascular Division, Faculty of Life Sciences and Medicine, The Rayne Institute, King’s College London, St Thomas’ Hospital, London, SE1 7EH UK

**Keywords:** Drug safety, Pharmacology

## Abstract

The ‘translational therapeutic index’ (TTI) is a drug’s ratio of nonclinical threshold dose (or concentration) for significant benefit versus threshold for adversity. In early nonclinical research, discovery and safety studies are normally undertaken separately. Our aim was to evaluate a novel integrated approach for generating a TTI for drugs intended for prevention of ischaemia-induced ventricular fibrillation (VF). We templated the current best available class 1b antiarrhythmic, mexiletine, using the rat Langendorff preparation. Mexiletine’s beneficial effects on the incidence of VF caused by 120 min regional ischaemia were contrasted with its concurrent adverse effects (on several variables) in the same hearts, to generate a TTI. Mexiletine 0.1 and 0.5 µM had no adverse effects, but did not reduce VF incidence. Mexiletine 1 µM reduced VF incidence to 0% but had adverse effects on atrioventricular conduction and ventricular repolarization. Separate studies undertaken using an intraventricular balloon revealed no detrimental effects of mexiletine (1 and 5 µM) on mechanical function, or any benefit against reperfusion-related dysfunction. Mexiletine’s TTI was found to be less than two, which accords with its clinical therapeutic index. Although non-cardiac adversity, identifiable from additional *in vivo* studies, may reduce the TTI further, it cannot increase it. Our experimental approach represents a useful early-stage integrated risk/benefit method that, when TTI is found to be low, would eliminate unsuitable class 1b drugs prior to next stage *in vivo* work, with mexiletine’s TTI defining the gold standard that would need to be bettered.

## Introduction

Coronary heart disease and acute myocardial infarction (AMI) constitute the largest cause of death worldwide^1^ with sudden cardiac death (SCD) responsible for approximately 50% of lethality^2,3^. Most SCD is due to ischaemia-induced arrhythmias, particularly ventricular fibrillation (VF) and ventricular tachycardia^4^.

Currently available antiarrhythmic drugs have failed against SCD^5^. The reason for this is that the benefit afforded by those drugs found to suppress SCD is offset by adverse drug reactions (ADRs); in some trials, mortality was actually increased^6–8^ while in other trials attempts to minimise ADR risk by use of lower drug doses resulted in loss of effectiveness^9,10^.

Presently the class 1b antiarrhythmic, mexiletine, and the non-selective class III drug, amiodarone^11^, are the only antiarrhythmics prescribed for SCD outside the hospital setting, but even these drugs are not considered sufficiently efficacious for use in the larger lower-risk populations^5,12^ leaving a huge and longstanding unmet therapeutic need^4^. Since amiodarone is an anomalous example of a class (III) that is otherwise discounted as a solution to SCD owing to proarrhythmia^5,12^, this leaves only class 1b agents as an avenue for new drug development.

An essential part of the process of any new drug development is to mitigate against ADRs during the nonclinical phase. In view of this, we and others^13^ advocate better characterization of the ‘translational therapeutic index’ (TTI) in nonclinical models before first in human (FIH) studies are attempted. A TTI can be defined as the ratio between the highest concentration without ADRs and the lowest concentration obtaining meaningful benefit, with a value of > 30 being deemed a minimum for most therapeutic scenarios^14^.

Our attempts to develop a new safer and more effective antiarrhythmic drug have recently focused on the testing of an ischaemia-selective class 1b drug, with preliminary data showing promise^15^. However, in order to gauge the potential advantage of this or any other new drug, we first need to formally define the gold standard: the template TTI of the best available current drug.

Mexiletine is prescribed for use against life-threatening ventricular arrhythmias^5,16^ with antiarrhythmic benefit mediated by block of sodium currents within the ischaemic region and termination of re-entrant conduction^17^. In addition to its widely documented *noncardiac* ADRs^18^, mexiletine also has the capacity to adversely increase atrioventricular (AV) conduction time^19–24^. It can also elicit an adverse negative inotropic effect *in vitro*^25,26^, *in vivo*^27,28^, and in the clinical setting^29,30^, albeit negative inotropic effects are not dose limiting^31–39^.

Despite its limitations, mexiletine is the only orally-active class 1b antiarrhythmic to have had widespread clinical exposure, so its TTI in a nonclinical disease model would provide the necessary template to allow comparison, and evaluation of the potential value, of any novel class 1b agent, by setting the height of the bar that any new drug must reach in order for it to become a candidate for translation.

A TTI in nonclinical research can be hard to obtain and harder to interpret owing to the multiplicity of experiments and models that may be used to generate different types of data (e.g., concentration-dependence of channel block, receptor affinity, pharmacokinetic data, and dose-dependence of effectiveness in a disease model) constituting the nonclinical portfolio. However, these obstacles diminish if readout for benefit and adversity are obtained at the same time using a single preparation (contiguously). If, in addition, studies are undertaken *in vitro* or *ex vivo*, complications associated with steady state drug concentration (affected by drug metabolism and distribution and plasma protein binding) can be disregarded and the TTI can be defined in terms of drug concentration rather than dose^13^.

Thus, in the present study we have characterized the TTI of mexiletine in the rat isolated heart, a standardized bioassay for detecting beneficial drug effects on ischaemia-induced VF^40^ and cardiac ADRs. The latter are manifest and defined as any effect on heart rate, coronary flow, intervals in the electrocardiogram (ECG), or ventricular mechanical function before the onset of, or during, ischaemia.

Using this integrated approach, we found that suppression of ischaemia-induced VF could *not* be obtained at any concentration devoid of one or more ADR. Given this, and the evidence that *noncardiac* ADRs are the threshold ADRs for mexiletine *in vivo* in humans^31,34,35,37–39^, it is evident that mexiletine has an unfeasibly narrow therapeutic window for suppression of ischaemia-induced VF (TTI < 2). We propose that, in future research, one may use the present data set as a template with which to gauge the potential value of new drugs, using an equivalent protocol.

## Methods

### Arrive, ethical, legal and experimental requirements

Animal housing and husbandry were as previously described^41^ and in compliance with ARRIVE^42^, the UK Home Office Guide on the Operation of the Animals (Scientific Procedures) Act 1986, and appropriate guidance on experimental design and analysis^43,44^. All experiments were performed under project licence PPL 70/7491 at King’s College London, and were approved by the King’s College London Animal Welfare and Ethical Review Board.

### Animals and general experimental methods and materials

Rats were chosen for studies because they have a coronary anatomy that lends itself to generation of reproducible susceptibility to ischaemia-induced VF that is well characterised, and the *in vitro* (Langendorff perfusion) variation lends itself to the tractable evaluation of the concentration-dependence of the effects of drugs on VF and other variables^40^. Male Wistar rats (254–475 g) were anaesthetised with a lethal dose of sodium pentobarbitone (170 mg.kg^−1^) and heparin (160 IU.kg^−1^) via intraperitoneal (i.p.) injection to achieve a surgical level of anaesthesia, confirmed by removal of pedal and corneal reflexes. Hearts were then excised (causing death by exsanguination) and immediately submerged in cold (4 °C) Krebs solution containing NaCl 118.5 mM, CaCl_2_ 1.4 mM, glucose 11.1 mM, NaHCO_3_ 25.0 mM, MgSO_4_ 1.2 mM, NaH_2_PO_4_ 1.2 mM and KCl 3 mM. Hearts were then cannulated via the aorta for Langendorff perfusion via the coronary arteries, and perfused with Krebs (as above), filtered (5μm pore size), warmed to 37 °C and gassed with 95% O_2_ and 5% CO_2_ to achieve a pH of 7.4, at a constant pressure of approximately 80 mmHg. Mexiletine (Sigma-Aldrich, UK) stock solutions were made up in a vehicle of 1:4, Ethanol (VWR International, UK): Water (PURELAB ELGA Process Water, UK). Krebs perfusate salts were purchased from VWR International, UK.

### Coronary artery ligation

A unipolar electrode was inserted in the apex of the heart, connected to a PowerLab (ADInstruments, UK), and used to detect heart rate and rhythm, with arrhythmias defined, detected and analysed according to guidance from The Lambeth Conventions^44^. A silk suture (4-0) was sewn around the left anterior descending (LAD) coronary artery, 1-2 mm below the left atrial appendage towards the apex, and threading both ends of the suture through a polythene tube. The suture was later tightened with and clamped (using forceps) to occlude the LAD coronary artery, inducing regional ischaemia, and subsequently loosened to reperfuse the artery. Following completion of the experimental protocol, the involved zone (IZ) size, defined as the anatomical region of myocardium that had been subjected to ischaemia, was identified by re-occlusion during perfusion with 5% disulphine blue dye (Patent blue VF sodium salt, Sigma-Aldrich, UK), dissected and quantified as % of total ventricular weight (TVW). An IZ size of 40-60% TVW was expected given the chosen suture positioning^45^.

### Experimental design and protocol

Mexiletine concentrations were chosen to span values slightly below and above reported clinically effective free plasma concentrations for suppression of ventricular arrhythmias^46,47^. Three concentrations of were examined: 0.1 μM, 0.5 μM and 1 μM (n = 18/group), all of which were compared with a matched vehicle control group (3 x n = 18). Initially, 1 µM was examined in the expectation this would suppress VF but possess ADRs. Once this was confirmed, the lower concentrations of mexiletine were studied in order to reveal at what concentration VF suppression could be achieved without ADRs. This experimental design was implemented to minimise animal usage. All experiments were blinded and randomised and followed a defined protocol.

Following a 5 minute period of Krebs perfusion, measurements of heart rate (beats.min^−1^), coronary flow (ml.min^−1^.g^−1^), PR interval and QT_90_ (QT interval at 90% repolarisation) interval times (msec) were begun, repeated at specified time points during the protocol. Perfusate was then switched to a test solution or control. After a further 5 minutes, regional ischaemia was induced for 120 minutes, followed by reperfusion for 10 minutes, and then IZ size delineation. In a subset of the hearts perfused with 1 μM mexiletine (n = 9) a higher K^+^ concentration of 5 mM K^+^ was introduced at the 90^th^ minute of ischaemia, with mexiletine or control solution continued for the remainder of the protocol, to examine whether any antiarrhythmic or adverse effects of mexiletine were dependent on ambient K^+^ in the heart, a factor examined previously and found to influence outcome in studies with other class I (and class IV) antiarrhythmics^48–50^. The 120 minute length of ischaemia was also chosen to capture beyond the scope of the full spectrum of phase 1 ischaemia-induced ventricular arrhythmias in the rat isolated heart, which arise and then dissipate during the first 90 minutes of regional ischaemia^51,52^. The occurrence of VF during the first 90 minutes of ischaemia was presented and analysed separately from VF occurring later because there is a possibility that mechanisms of VF differ between early and more sustained ischaemia, and the actions of drugs diminished, exacerbated or even converted from protective to proarrhythmic^51,53^. Our protocol was designed to explore all these possibilities without adding unnecessary extra groups (minimising animal usage).

### Coronary flow and ECG analysis

Coronary flow (ml.min^−1^.g^−1^) was measured by timed collection of effluent, which was weighed (1 g = 1 ml), and then expressed relative to the TVW of the respective heart. ECG and pressure measures such as heart rate (beats.min^−1^), PR and QT_90_ intervals (msec), diastolic and developed pressures (mmHg) were obtained only during sinus rhythm. Ventricular arrhythmias defined according to the Lambeth Conventions II^44^ were recorded as ‘present’ or ‘absent’ within sequential 30-minute time periods during ischaemia, and the during first 10 minute period of reperfusion.

### Exclusion criteria

Any heart that, prior to switch to test solution, had a coronary flow that fell outside the range of 7 to 20 ml.min^−1^.g^−1^, a sinus heart rate of less than 200 beats.min^−1^, or an IZ size less than 40% TVW was excluded from the study, and subsequently replaced to maintain equal group sizes while maintaining blinding^41^. Hearts that met exclusion criteria were not excluded if doing so would not affect the outcome of the study (i.e. if VF occurred despite the IZ being below the size criterion, which is used to ensure that if VF did not occur in an individual heart, it was not due to IZ being ‘too small for VF to occur’^54^).

### Supplementary studies assessing left ventricular contractile function

Evaluation of left ventricular function was undertaken using Krebs modified to contain 4 mM KCl in an experiment undertaken separately from the arrhythmia study, owing to technical reasons recently identified^55^. A deflated intraventricular balloon (IVB), made from complaint non-elastic material, attached to a pressure transducer was inserted into the left ventricle via the left atrium for assessment of two components of contractile function, namely diastolic function as defined by diastolic pressure, and inotropy as defined by developed pressure (the difference between peak systolic pressure and minimum diastolic pressure during a single cardiac cycle during sinus rhythm). Once attached to the perfusion apparatus, hearts were submerged in coronary effluent contained within a heated jacket (37 °C) to preclude a fall in temperature, later, during global ischaemia. The IVB was filled with a small volume of saline (~0.02 ml) so that a pressure could just be measured, and the arbitrary value on the syringe noted as the ‘zero volume’. Using the zero volume as a reference point the syringe was then filled with a volume of saline (typically ~0.12 ml for a 0.7–0.9 g heart) sufficient to generate a developed pressure >100 mmHg, without elevating diastolic pressure to >10 mmHg (avoiding excessive diastolic stretch). This volume was defined as the ‘working volume’ and was set when examining effects of solution switching and effects of ischaemia and reperfusion. After cessation of each protocol, the hearts were removed from the Langendorff set up, the atria and any extra-cardiac tissue was removed, and the TVW (g) was measured.

#### Experimental design and protocol

Mexiletine was evaluated at up to 5 μM on the expectation based on clinical reports that adverse effects on contractile function would require a higher concentration than that evoking adverse effects on the ECG^38^. Each group was compared with a vehicle control group (all n = 9). Following a 5 minute period of Krebs perfusion, measurements of heart rate (beats.min^−1^), coronary flow (ml.min^−1^.g^−1^), ventricular diastolic pressure (mmHg) and developed pressure (mmHg) were taken at various predetermined time points. A Starling curve was constructed by deflating the IVB to zero volume before inflating the IVB with 0.02 ml increments of saline (up to a diastolic pressure <20 mmHg) and recording the incremental changes in diastolic and developed pressure (mmHg). Perfusate was then switched to one of the three test solutions and 5 minutes later a second Starling curve was constructed. Global ischaemia was then induced by clamping the tubing above the aortic cannula, maintained for 20 minutes. Reperfusion was initiated by releasing the clamp, using the same test solution, and maintained for 60 minutes, whence a third Starling curve was constructed to determine drug effects on recovery of mechanical function.

### Data and statistical analysis

Selection of group sizes was based on previous experience with the models used^40^, and an expectation that assessment of threshold concentrations achieving effects (i.e., detection of weak drug effects) would require larger group sizes than normal for establishing statistical significance, as found in previous studies^41^. Studies were undertaken using randomization followed by blinded analysis^44^ and in accordance with BJP’s guidance in terms of group sizes, normalizations (none used) and Y axis labelling (all raw variables and no ‘fold control’)^43^. Statistical analysis was undertaken to determine whether individual test groups differed from drug-free controls. Gaussian distributed variables were subjected to an unpaired t test (when there were only two groups) or to ANOVA (1 way or 2 way, repeated measures when appropriate) when more than two groups were contrasted. After ANOVA, if F was significant and no variance inhomogeneity was detected, Dunnett’s *post hoc* test was used to identify the groups in which variables had changed compared with controls. All Gaussian-distributed values were expressed as mean ± SEM since representation of raw data values or standard deviations renders many data sets impossible to fathom due to clutter. Binomially distributed variables were compared using Fisher’s exact tests. The threshold P value denoting statistical significance was P < 0.05. Differences between groups are described as effects or changes always and only when statistically significant. Statistical analysis was performed in GraphPad prism 7 (UK) software.

### Ethical approval

All applicable international, national, and/or institutional guidelines for the care and use of animals were followed.

### Declaration of transparency and scientific rigour

This paper adheres to the principles for transparent reporting and scientific rigour of nonclinical research recommended by funding agencies, publishers and other organisations involved with supporting this research.

## Results

### Design validation

**Figure 1:**
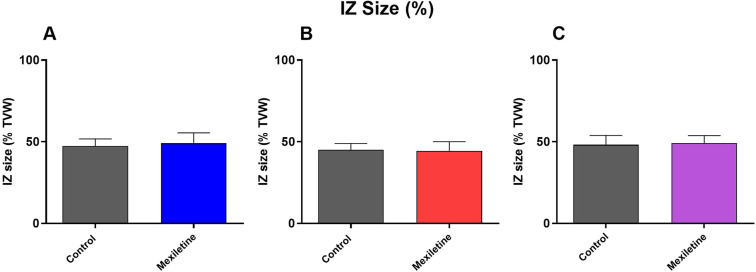
Involved Zone Size (% TVW) resulting from proximal ligation of the left coronary artery in rat isolated hearts perfused with (**A**) 0.1 µM mexiletine, (**B**) 0.5 µM mexiletine and (**C**) 1 µM mexiletine, measured as % total ventricular weight (TVW) each compared with separate groups of matched control hearts, n = 18 per group, mean ± SEM. There were no statistically significant differences between groups.

IZ size was similar in each group (Fig. 1A–C), validating the effectiveness of randomisation in fulfilling the requirement for parity of arrhythmogenic stimulus^54^.

### Concentration-dependence of effect of mexiletine on ischaemia-induced VF

**Figure 2:**
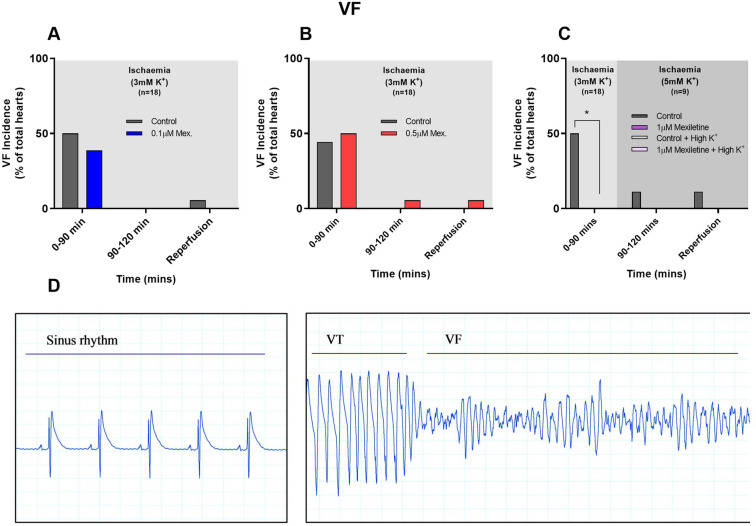
Incidence of Ventricular Fibrillation (% of total hearts per group) during 120 min of regional ischaemia, and (separately) during 10 min reperfusion, for rat hearts perfused with or without (**A**) 0.1 µM mexiletine, (**B**) 0.5 µM mexiletine and (**C**) 1 µM mexiletine (here, in half of the hearts, the K^+^ in the solution was changed 30 min before the start of reperfusion to 5 mM with all other constituents including drug concentration unchanged), n = 18 per group, and n = 9 (for the last 30 min of ischaemia and 10 min reperfusion). *P < 0.05 versus control. (**D**) Representative ECG traces displaying sinus rhythm, ischaemia-induced VT and ischaemia-induced VF during first 30 mins of regional ischaemia.

The incidence of VF was approximately 50% in controls, providing scope for detection of inhibition by mexiletine (Fig. 2A–C). Mexiletine had no effect on VF at 0.1 and 0.5 µM whereas 1 µM abolished it, indicating a steep concentration-dependence (Fig. 2A–C). Representative ECG traces of sinus rhythm, ischaemia-induced VT and ischaemia-induced VF are displayed in Fig. 2D.

### ADRs

**Figure 3:**
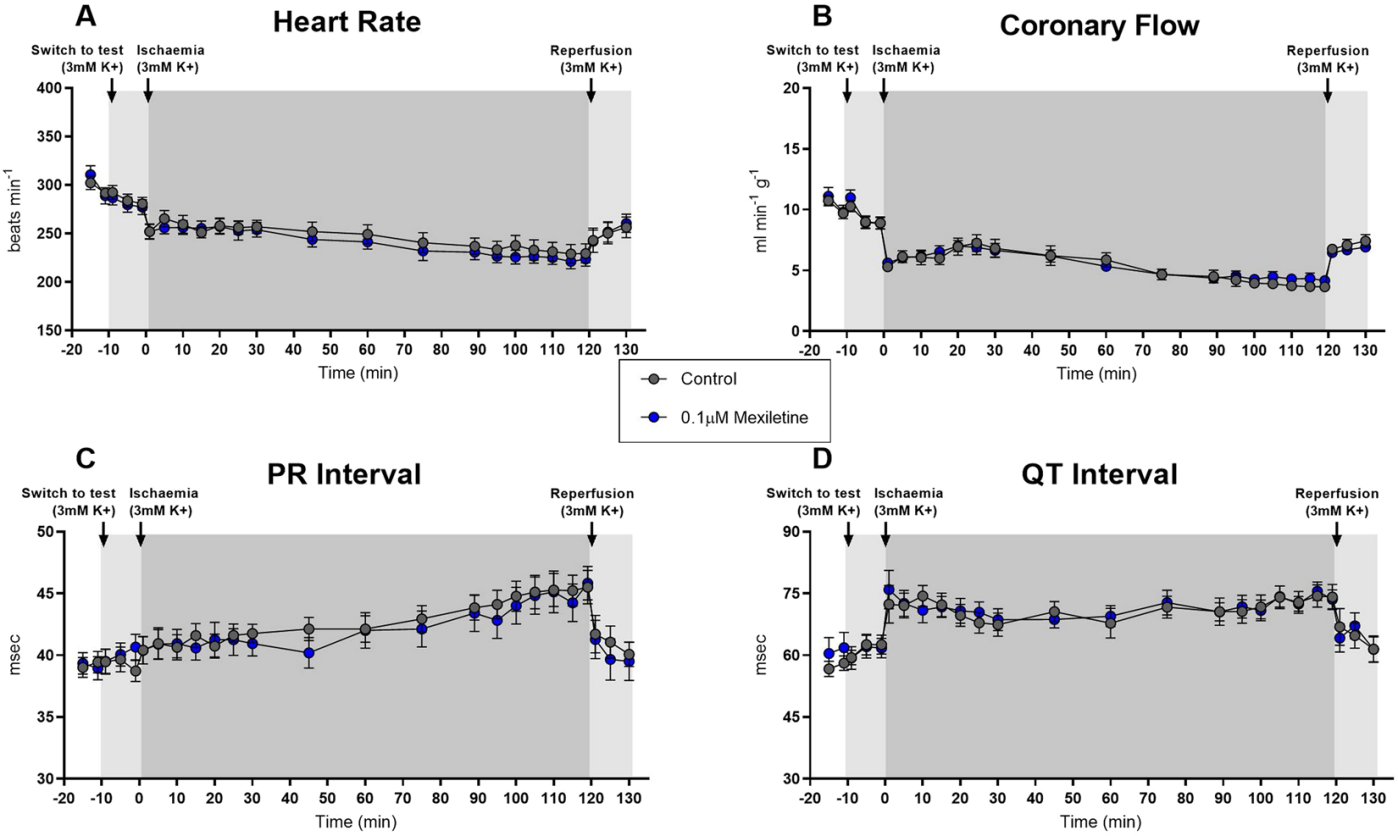
Haemodynamic and ECG changes in rat hearts perfused with or without 0.1 µM mexiletine during 120 minutes left coronary artery ligation and 10 minutes reperfusion. (**A**) Heart rate (beats.min^−1^), (**B**) Coronary flow (ml.min^−1^.g^−1^), (**C**) PR interval (msec), (**D**) QT_90_ interval (msec). n = 18 per group; mean ± SEM. *P < 0.05 versus time-matched vehicle control. There were no statistically significant differences between groups for any variable.

**Figure 4:**
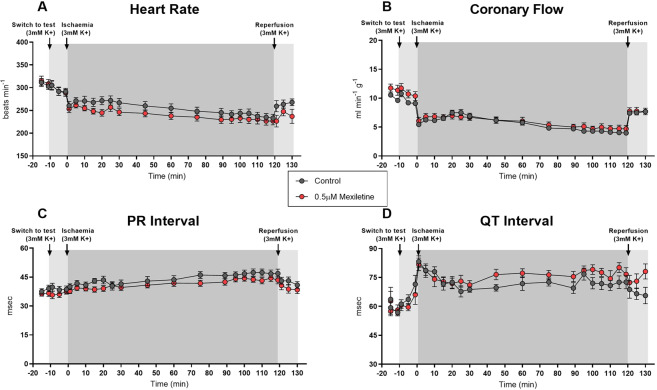
Haemodynamic and ECG changes in rat hearts perfused with or without 0.5 µM mexiletine during 120 min left coronary artery ligation and 10 min reperfusion. (**A**) Heart rate (beats.min^−1^), (**B**) Coronary flow (ml.min^−1^.g^−1^), (**C**) PR Interval (msec), (**D**) QT_90_ Interval (msec). n = 18 per group, mean ± SEM. *P < 0.05 versus time-matched vehicle control. There were no statistically significant differences between groups for any variable.

**Figure 5:**
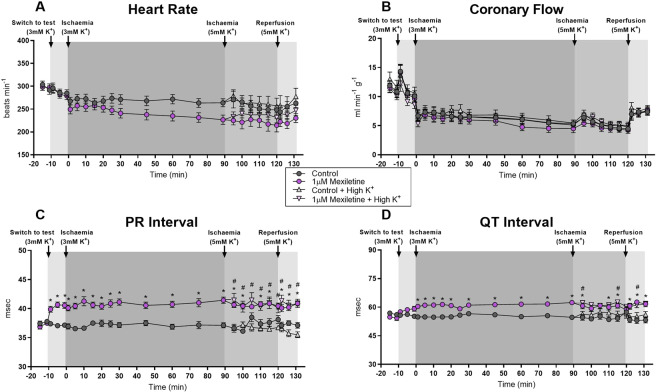
Haemodynamic and ECG changes in rat hearts perfused with or without 1 µM mexiletine during 120 min left coronary artery ligation and 10 min reperfusion. Here, in half of the hearts, the K^+^ in the solution was changed 30 min before the start of reperfusion to 5 mM with all other constituents including drug concentration unchanged, n = 18 per group, and n = 9 for the last 30 min of ischaemia and 10 min reperfusion. (**A**) Heart rate (beats.min^−1^), (**B**) Coronary flow (ml.min^−1^.g^−1^), (**C**) PR Interval (msec), (**D**) QT_90_ Interval (msec). Values are mean ± SEM. *P < 0.05 mexiletine versus time-matched vehicle control, ^#^P < 0.05 mexiletine + high K^+^ versus time-matched high K^+^ vehicle control.

Recorded variables in control hearts followed the pattern typical for the preparation^51,55–57^, with heart rate and coronary flow falling slightly during ischaemia, and then recovering during reperfusion, PR intervals varying little over time, and QT intervals increasing during ischaemia and decreasing during reperfusion (Fig. 3, 4 and 5A–D). Neither 0.1 nor 0.5 µM mexiletine had any effect on any variable (Fig. 3A–D). 1 µM mexiletine did not affect coronary flow (Fig. 5B), or heart rate versus control (Fig. 5A), but it did prolong the PR interval and QT interval with effects beginning before the onset of ischaemia (Fig. 5C,D), indicating off-target pharmacology in non-ischaemic supraventricular and ventricular tissue. The introduction of 5 mM K^+^ at the 90th minute of ischaemia did not alter these effects.

### Effects of mexiletine on left ventricular contractile function

**Figure 6:**
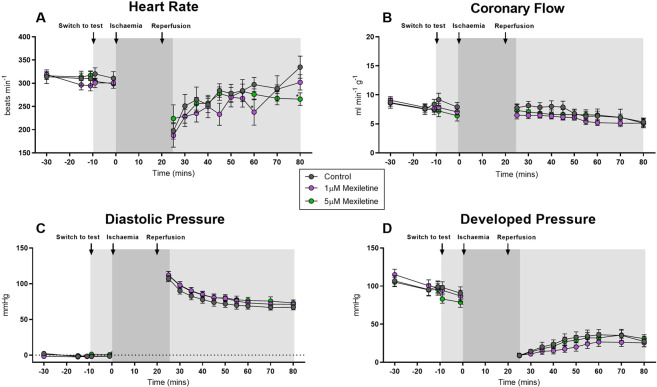
Haemodynamic and ECG changes during 20 min global ischaemia and 60 min reperfusion in rat hearts perfused with or without 1 µM mexiletine or 5 µM mexiletine. (**A**) Heart rate (beats.min^−1^), (**B**) coronary flow (ml.min^−1^.g^−1^), (**C**) diastolic pressure (mmHg) and (**D**) developed pressure (mmHg). All n = 9 per group, mean ± SEM. *P < 0.05 versus time-matched vehicle control. There were no statistically significant differences between groups for any variable.

Mexiletine, tested only at the higher two concentrations (1 µM and 5 µM), had no effect on heart rate or coronary flow (Fig. 6A,B), replicating findings (above) from hearts not fitted with an IVB. Prior to ischaemia, neither concentration of mexiletine affected diastolic or systolic pressure at the IVB’s working volume (Fig. 6C,D). In all groups, diastolic pressure was increased, and developed pressure was decreased during 60 min reperfusion following global ischaemia with the partial recovery of function during reperfusion not altered by 1 or 5 µM mexiletine (Fig. 6C,D).

**Figure 7:**
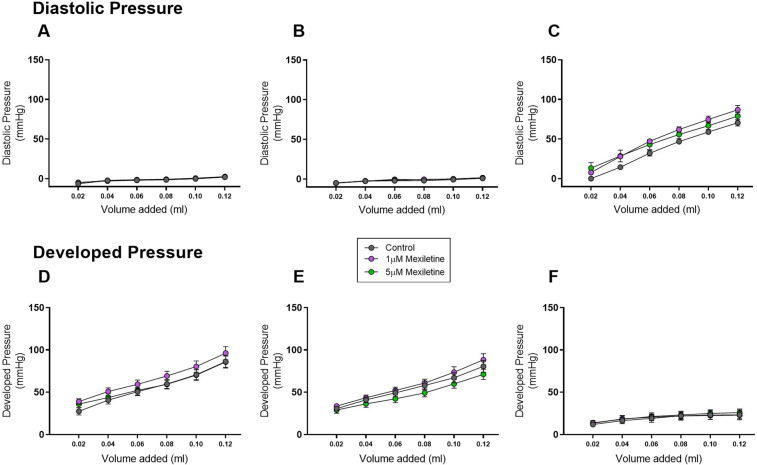
Changes in Diastolic pressure (mmHg) and Developed pressure (mmHg) generated from Starling curves in the rat Langendorff preparation, with or without 1 µM mexiletine and 5 µM mexiletine performed during Krebs perfusion (**A,D**), after switch to 1 µM mexiletine, 5 µM mexiletine or vehicle (**B,E**) and following 20 min global ischaemia and 60 min reperfusion (**C,F**). The slope of diastolic or developed pressure (mmHg) and IVB volume (ml) was analysed by linear regression analysis. All n = 9 per group, mean ± SEM. There were no statistically significant differences between groups for any variable.

Consistent with these findings, Starling curve analysis revealed that neither 1 µM nor 5 µM mexiletine had any discernible effect on either systolic or diastolic function prior to ischaemia, or during reperfusion, with the slope of each volume:pressure line indistinguishable from control throughout the protocol (Fig. 7A–F).

## Discussion

### Mexiletine and the need for quantitative nonclinical therapeutic window data

Among the few drugs used to treat severe life-threatening ventricular arrhythmias during, or following, AMI^5,58^ mexiletine is the archetypal orally-active class 1b antiarrhythmic^11^. It is the only clinically used antiarrhythmic for prevention of SCD that represents an archetype that may serve as a gold standard, since the only other drug for this indication, amiodarone, is anomalous within its nominal class^5^, and not an archetype that may serve as a focus for novel drug development. Clinically effective plasma mexiletine concentrations that provide antiarrhythmic benefit without treatment-terminating ADRs are reported to be 0.5-2.0 µg/ml^46,47^. To achieve this, an oral dose of 200-300 mg every 8 hours is required [16]. However, the plasma concentration range of a drug encountered therapeutically is often wider than the therapeutic window^13^. The average plasma concentration of mexiletine causing ADRs is 0.88 µg/ml, and the threshold concentration triggering treatment withdrawal is approximately 0.95 µg/ml^38^. The clinical therapeutic window is thus concerningly narrow. Unsurprisingly therefore, although initial clinical trials reported positive findings^31–35,37,39,59^ mexiletine has not emerged as a drug for primary prevention of SCD^12^; as with all antiarrhythmics its ADRs are perceived to outweigh benefit in this context^5,16^. Consequently, since this, the most useful drug has limited utility, there remains an important unmet clinical need for primary prevention.

In mexiletine’s nonclinical development phase it would have been useful to have had a clear characterization of the doses and concentrations obtaining *safe* antiarrhythmic activity. Currently, for a drug to be viable for advancement to therapeutic evaluation in humans it must have a an adequate TTI^13^, and the drug with the best TTI would serve as a template with which to judge developing drugs. Unfortunately, although mexiletine was examined for antiarrhythmic activity in a variety of animal models *in vitro*^60^, *ex vivo*^61,62^ and *in vivo*^27,63–66^, no systematic attempt was made to determine its TTI. The aim of the present study was to rectify this, using the highly tractable rat Langendorff isolated perfused heart model and, in doing so, exemplify the model/approach and provide a mexiletine data set for use as a template with which to evaluate whether novel, as yet, untested drugs offer an advantage.

### Principal findings and implications

We found that 0.1 µM and 0.5 µM mexiletine did not exhibit antiarrhythmic effects. Unsurprisingly, these concentrations are lower than those (in plasma) reported to provide clinical effectiveness^46,47^. A doubling of concentration to 1 µM was effective against VF. However this concentration precipitated ADRs, causing prolongation of the PR and QT intervals, just as a doubling of plasma concentration to 1 µg/ml results in serious ADRs in humans^38^, and subtherapeutic doses of mexiletine in a conscious rat model of ischaemia-induced VF caused an unacceptable reduction in blood pressure^67^. These results indicate that the consensus therapeutic index for mexiletine in humans (based on disparate data) was replicated by the TTI in the rat Langendorff preparation, and was confirmed to be impracticably small, requiring a concentration above 0.5 µM yet below 1 µM - a TTI of less than two. This is clearly insufficient to meet the 30-fold criteria typically predictive of a safe, effective drug^14^.

Overall these findings validate the experimental approach elaborated in the present study and explain the limited clinical utility of mexiletine.

### Study strengths and limitations

The Langendorff-perfused rat heart has been used extensively for evaluating drug-induced effects on ischaemia-induced VF, and is highly tractable^40^. Additionally, it provides scope for identifying drug-induced ADRs for numerous drug classes including those that block cardiac sodium current (I_Na_) and cardiac and vascular L-type calcium current (I_CaL_)^40,49,50,53,57,68^ albeit not delayed rectifying potassium current blockers^40^. In addition to detection of ECG effects, increases in coronary flow in the perfused rat heart^50,69^ are predictive of peripheral vasodilatation and clinical hypotensive effects^70^. The present approach therefore allows early identification of a new drug as unviable owing to dose limiting cardiac ADRs. However, an isolated perfused heart self-evidently cannot be used to assess noncardiac ADR risk.

Concern about possible noncardiac ADRs would arise if a drug were found to have a TTI in the rat isolated heart that is sufficiently large to justify further nonclinical development (i.e., >30), and noncardiac ADRs would then be examined by other means. The advantage of our approach, however, is that any drug *with* cardiac ADRs will have been excluded from further development. Thus, although mexiletine can cause central nervous system toxicity and gastrointestinal discomfort at plasma concentrations lower than those causing ADRs related to the ECG^31,34,35,37–39,71^, its small TTI in the rat isolated heart renders its extracardiac ADRs irrelevant. For any new class 1 antiarrhythmic, a TTI of >30 would need to be met using our protocol, and only then would examination of its noncardiac ADRs be justified; reducing animal usage is an added benefit.

The rat heart has numerous advantages for TTI assessment of putative anti-VF drugs that are well documented, including an ability to manifest a reproducible susceptibility to ischaemia-induced VF^40^, but some hypothetical reasons for concern about this species choice remain. Class 1 drug-induced ventricular or AV nodal conduction abnormalities may occur to a greater extent, or at a lower drug concentration, in species such as the rat that has a basal heart rate higher than that of humans, if the drug’s effects on I_Na_ are characterized by slow binding and unbinding rates, typical of class 1c and 1a drugs^72^. However, this would not be a concern for drugs that bind and unbind from sodium channels quickly, such as mexiletine and other 1b drugs^72^. The present data accord with this since mexiletine’s TTI in the rat heart mapped to the human therapeutic index. The predictive value of the rat heart may not be optimal for class 1c or 1a drugs, but these drugs are no longer used for treating ventricular arrhythmias, so the need to test a novel 1c or 1a drug in *any* nonclinical model of SCD and VF is unlikely to arise.

### Conclusions

Nonclinical data sets can be complex with a variety of estimates of affinity and potency for actions on a range of different molecular targets and tissues. Additionally, safety and effectiveness are rarely investigated in tandem. This makes it difficult to obtain an estimate of a TTI. For a decision to be made on the likely clinical success and safety of a new antiarrhythmic, the nonclinical database must be sufficiently accurate to mitigate against false inference. In the SCD therapeutic area, failed translation has become the rule rather than the exception, with development blighted by low effectiveness coupled with ADRs at therapeutic dosage^6–10^. This requires better *early-stage* elaboration of the TTI using simple tractable approaches.

The integrated evaluation of safety and effectiveness (exemplified in the present study) aims to reduce animal requirements by effectively halving the number needed to estimate the TTI if effectiveness and safety were evaluated independently. The rat Langendorff preparation was shown to detect cardiac ADRs contiguously with beneficial effects on ischaemia-induced VF with a TTI (<2) that replicates the consensus clinical therapeutic index. The experimental approach outlined was therefore validated and provides a template ‘gold standard’ for future early nonclinical assessment of novel antiarrhythmics, particularly antiarrhythmics with class 1b characteristics, the one remaining class that is not intrinsically flawed owing to insurmountable ADRs including proarrhythmia related to its primary molecular pharmacology in the heart.

Finally, it is important to clarify the context of the present work. If a novel drug (class 1b, certainly, or drug of novel class), were to be found to have a promising therapeutic index in the rat Langendorff preparation this would allow its development to proceed, and if the drug were later found to have dose-limiting *noncardiac* adversity this will be detected by other means, for example when the efficacy and safety are examined using an *in vivo* animal model. Species with a lower basal heart rate than the rat, such as the dog^73^ are not suitable for *early* nonclinical evaluation owing to costs and ethical considerations. Our suggestion would be to utilise the Langendorff-perfused rat heart model to benchmark the TTI of a class 1b agent and verify findings *in vivo*, and then in a second species, only if justified at each stage of experimentation (TTI > 30-fold). Class 1b drugs that *do* have dose-limiting cardiac ADRs that give rise to a narrow TTI *will* be detected in the rat Langendorff preparation, and rejected, saving the animals, time and cost that would have been expended exploring the drug’s ADRs by other means.
